# Cornea Update: An Overview of the Topics Presented in this Issue

**DOI:** 10.4103/0974-9233.61209

**Published:** 2010

**Authors:** Majid Moshirfar

**Affiliations:** Department of Ophthalmology, Salt Lake City, UT, USA

Every issue of MEAJO represents an extraordinary confluence of hundreds of hours of human efforts. In this issue of MEAJO, authors with expertise in the area of corneal and external disease as well as laser refractive surgery have sincerely undertaken their task to the best of their capacity and published remarkable reviews for our readers.

For over a century, ophthalmologists have been treating Fuchs dystrophy and bullous keratopathy with penetrating keratoplasty. Now, in the last decade, we have witnessed the groundbreaking alter in the treatment of corneal endothelial diseases. In this issue, Dr. Natalie Afshari and associates from Duke University, North Carolina, have provided our readers an excellent overview of modern endothelial keratoplasty (EK) and its surgical evolution from the time when Gerrit Melles set the groundwork in a procedure he called posterior lamellar keratoplasty (PLK). Each surgical modification of EK, from PLK to deep lamellar endothelial keratoplasty and further refinement to Descemet's stripping automated endothelial keratoplasty to the newest version of EK, known as Descemet's membrane endothelial keratoplasty (DMEK), reflects the ever more selective transplantation of corneal endothelial cells. I am certain that in the next several years, further refinement of DMEK and endothelial cell tissue harvesting will bring not only excitement but also an explosion of better surgical techniques in the field of EK.

On another note in the recent years, the excitement concerning the use of *ex vivo* expanded bioengineered epithelial on amniotic membrane as the carrier tissue for partial and total stem cell disease without the risk of rejection has brought tremendous hope to many ophthalmologists who have dealt with patients suffering from limbal stem cell deficiency with severe corneal neovascularization and opacification. In this issue, Dr. Ali Djalilian and his associates from the University of Illinois at Chicago not only provide us with a comprehensive description of the surgical technique for limbal stem cell transplantation (LSCT) but also, more importantly, address the importance of ocular surface optimization and control of inflammation prior to LSCT in order to increase the chances of graft survival. In addition, authors have performed a superb job of providing our readers a relevant but extremely concise medical regimen of systemic and topical immunosuppressants for all allograft recipients.

In this issue, several newer, non-traditional treatments for keratoconus have also been addressed. Dr. Ladan Espandar from Tulane University and Dr. Michael J. Meyer from South Carolina University initially discuss the intrastromal corneal ring segments (ICRS), Intacs severe keratoconus, along with a new Ferrara ring with a 210-degree arc single segment. They continue to discuss the clinical application of various phakic intraocular lens (IOL) for the correction of refractive error after implantation of intrastromal rings. Dr. Mirko R. Jankov II from Belgrade, Serbia, and associates have presented one of the most comprehensive review articles on the photooxidative collagen cross-linking technique using riboflavin and ultraviolet-A (UVA) light. The future management of keratoconus will likely focus on stabilizing the biomechanically weakened collagen of keratoconus, reshape and normalize the topography of the cornea, correct high refractive error and prevent progression of ectasia by incorporating the application of multiple treatment modalities such as collagen cross linking (CXL), ICRS, topography-guided photorefractive keratectomy (PRK) and phakic IOLs, either simultaneously or sequentially.

With the re-emergence of the old abandoned lamellar keratoplasty among ophthalmologists for better globe integrity as well as the elimination of endothelial rejection, Professor Farid Karimian and associates from Shahid Beheshti University in Tehran have provided us with a comprehensive review article discussing the indications, various surgical techniques and the clinical results along with the management of deep anterior lamellar keratoplasty (DALK) complications in this issue. Such increased popularity among ophthalmic surgeons can only be attributed to the recent improvements in better instrumentation as well as refinement of the surgical techniques available for DALK. 

This come at a time when corneal opacity remains the third leading cause of blindness in the developing world. Dr. Michael Feilmeyer and Dr. Geoffrey Tabin from the University of Utah have done a remarkable job addressing the use of Glycerol-preserved corneas in this issue. Authors emphasize the lack of eye banking and shortage of suitable tissue as some of the main limiting factors to sight-restoring keratoplasty in such countries. They continue to address the great interest in the potential use of acellular corneal tissue with the recent popularity and advancements in lamellar keratoplasty as a potential solution to corneal blindness provided that adequate surgical skill is available.

Professor Hassan Hashemi and associates from Tehran University have given us a practical overview of two of the very commonly used computerized topography systems in recent years: The Orbscan (Bausch and Lomb) and the Pentacam (Oculus), which are known as a slit-scanning device and a Schiempflug imaging device, respectively. By providing a day to day clinically relevant information and list of topographic and tomographic parameters, ratio and ranges, authors have provided us useful information for better screening of patients interested in refractive surgery, and perhaps some of the risk factors that would not make them suitable candidates for surgery. No doubt, further improvements in such diagnostic devices in terms of image acquisition and refinement in the artificial intelligence behind their sofwares will improve the sensitivity and specificity of such devices.

In the arena of refractive surgery, Laser *in situ* kerato mileusis (LASIK) continues to be the most popular refractive surgery procedure worldwide. Again, Dr. Ladan Espandar from Tulane University and her associates have discussed the rate and various type of intraoperative and post-operative flap complications using the mechanical microkeratome and Femtosecond laser technology. Authors continue to emphasize that despite Femtosecond laser gaining popularity in terms of safety and thickness predictability, flap complication has been reported with either technology in the literature. Espandar *et al*. have tried to compare and contrast various types of flap complications and address the rate and prognosis of each complication when using these two modalities for flap creation. As LASIK surgeons, we need to be aware of such complications and how to prevent them and have the knowledge to recognize and manage them in a timely manner for the best possible visual outcome.

The mysterious central toxic keratopathy (CTK) disease as a complication of LASIK and PRK surgery has definitely regained additional awareness among the refractive surgeons in the last several years. Even though Fraenkel *et al*. first described it over 20 years ago, until recently, CTK has been combined with the same disease spectrum as diffuse lamellar keratitis. Dr. Ribhi Hazin from Massachusetts Eye and Ear Infirmary and his associates have provided us an organized literature review of this disease process. As mentioned by the authors, there is a lack of agreement among clinicians, both in terms of accurately diagnosing CTK as well as in the best medical management for this condition. No doubt, further research involving examination of eyes with CTK using confocal microscopy and advanced computerized tomography may increase our understanding about the true nature of CTK and, thus, better treatment options based on objective findings.

Last, but not least, Dr. Vahid Feiz from the University of California, Davis, known for his great contribution to the Feiz-Mannis formula, has provided a quick overview concerning IOL calculation after cataract surgery. With the advancement of the computerized topography systems in recent years, such as slit-scanning devices and the Scheimpflug imaging tomography systems, we are closer than ever to obtaining the true corneal power regardless of the refractive status of the cornea. However, the accuracy and applicability of these true corneal power measurements have not been established clinically on a large scale. Despite the increasing number of methods to avoid IOL miscalculation after cataract surgery, we should continue to inform our patients who have had previous corneal refractive surgery of the limitations in accurate IOL power calculations and specifically discuss the possible need for corrective glasses, additional corneal refractive surgery or even IOL exchange.

It was sincerely a great honor for me to write this editorial for the cornea issue of the *Middle East Africa Journal of Ophthalmology* (MEAJO). As the new year began, I contemplated on the progress of last year and the great achievement of making our journal a Pubmed peer-reviewed scientific reference. I would also like to thank our contributing authors, reviewers and our dedicated editorial staff across the world, and continue to solicit our readers’ feedback on how to make MEAJO better in the future.

